# Description of Transperineostomal Resection of the Prostate: A Case Report

**DOI:** 10.7759/cureus.65026

**Published:** 2024-07-21

**Authors:** Luca Funk, Lukas John Hefermehl, Severin Hofmann, Anna Nikitin, Uwe Bieri

**Affiliations:** 1 Department of Urology, Kantonsspital Baden, Baden, CHE

**Keywords:** urethral stenosis, fournier’s gangrene, perineal urethrostomy, tur-p, lower urinary tract symptoms

## Abstract

We report a case of transperineostomal bipolar resection of the prostate (TPR-P) for lower urinary tract symptoms (LUTS). To our knowledge, this is the first description in the scientific literature. A 67-year-old man with a medical history of multiple penile debridements and formation of a perineostomy due to an episode of severe Fournier’s gangrene in 2015, was admitted to the emergency room with acute urinary retention. Consecutively, a suprapubic catheter was inserted. Attempts of catheterization failed due to bulbar stenosis and an obstructive prostatic urethra. After the resolution by dilatation of the bulbar stenosis, post-voiding residual volume persisted at up to 150 ml. The intra- and postoperative course after TPR-P was uneventful. No adverse events occurred. The assessment after six weeks revealed an International Prostate Symptom Score (IPSS) improvement of nearly 50% for the symptoms and >60% for overall satisfaction (preoperative: IPSS: S=24, L=6; postoperative IPSS: S=13, L=2). The average post-voiding residual volume decreased from 150 ml preoperatively to 15 ml (range 0-30 ml) postoperatively. Due to the missing full length of the urethra, the augmented range of motion seemed almost too loose for classic resection techniques in our hands. Therefore, we believe that in such cases it might be advantageous to use enucleation techniques. However, in our case, TPR-P was feasible and safe with a good functional outcome.

## Introduction

A perineostomy, also described as a perineal urethrostomy, is usually performed when the natural urinary passage cannot be restored to enable voiding through the meatus of the penis. Following the creation of a new urine exit point, males adopt a seated urination posture as urine exits the body via the perineal opening. Indications to perform a perineostomy include complex or recurrent urethral strictures (e.g. after Fournier’s gangrene) in patients not fit to undergo complex surgery [1], penectomy [2], hypospadias, or failed previous urethroplasty [3-5]. In our case, the patient suffered from Fournier’s gangrene.

With an incidence of 1.6 cases per 100,000 men per year, Fournier’s gangrene is a relatively rare disease. It is characterized as a necrotizing fasciitis that typically arises from infections affecting the urogenital tract, anorectal region, and genital skin [6]. It can extend fulminantly, leading to multiple organ dysfunction, septic shock, and death. Emergency treatment includes surgical removal of affected tissue in combination with broad antimicrobial therapy. As a result of the surgical debridement, in this case, the reconstruction of the penile urethra was not possible.

Non-neurogenic male lower urinary tract symptoms (LUTS) is a descriptive term relating to various underlying causes, including urethral strictures or benign prostatic obstruction. The management of male LUTS depends on the severity of symptoms, measured by the International Prostate Symptom Score (IPSS) [7]. Mild symptoms can be managed conservatively. Moderate symptoms are mostly treated pharmacologically [8]. Αlpha-1-adrenoceptor antagonists are used for voiding symptoms, and muscarinic receptor antagonists are recommended for dominant storage symptoms [8]. Surgical treatment should be considered if pharmacological treatments fail or symptoms become more severe [8].

Transurethral resection of the prostate (TUR-P) is the current standard surgical procedure for men with moderate prostate enlargement (30-80 ccm) and an IPSS between S=8 and 25, which corresponds to moderate to severe LUTS [7]. The procedure is performed with a rigid resectoscope through the urethra. Since TUR-P for larger prostates (>60 ccm) is associated with higher blood transfusion and revision rates, other techniques are recommended for more advanced prostate enlargement. In this regard, endoscopic laser enucleation techniques have a more favorable profile concerning bleeding and length of hospital stay compared to open or laparoscopic surgery [9].

To our knowledge, no transurethral bipolar resection of the prostate through a pre-existing perineostomy has yet been described in the contemporary scientific literature.

## Case presentation

A 67-year-old man presented with a medical history of multiple genital debridements and the forming of a perineostomy due to severe Fournier gangrene in 2015. After recovery, spontaneous voiding through the perineostomy was satisfactory for the patient, until presentation with acute urinary retention (AUR) at the emergency room in July 2023. Ultrasound confirmed the diagnosis of AUR with a residual volume (RV) of 500 ml in the urinary bladder. The kidneys showed no hydronephrosis. Prostate volume was not measured at that time. The attempt to insert a transperineostomal indwelling Foley catheter failed, and a 12 French suprapubic catheter was inserted. Urine analysis showed slightly elevated leukocytes, nitrite positivity, and the growth of *Klebsiella pneumoniae*.

**Figure 1:**
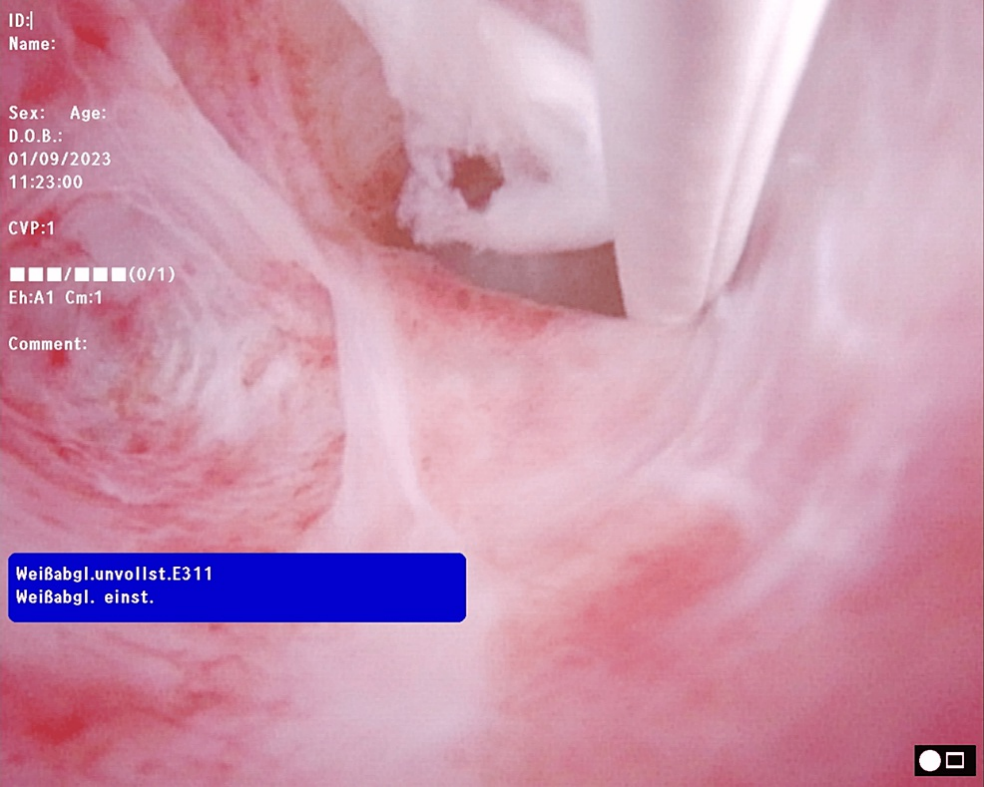
Subtotal stenosis of the bulbar urethra, a white guide-wire through the true lumen, and a small false passage in the left part of the figure

Differential diagnoses for AUR include lower urinary tract infections, subvesical obstruction, and iatrogenic (medication) and neurogenic conditions [10]. In our case, a lower urinary tract infection, most likely due to subvesical obstruction, was diagnosed and treated with trimethoprim/sulfamethoxazole according to the resistance profile. Two weeks later, voiding through the perineostomy was re-established by clamping of the suprapubic catheter. However, the patient reported a weak urinary flow, and an elevated post-voiding RV of 140-210 ml was recorded. Flexible urethrocystoscopy showed a small false passage due to previous attempts of catheterization and an unpassable subtotal stenosis of the bulbar urethra (Figure 1). A guidewire-assisted dilatation of the bulbar urethra using catheter sizes from 12 to 18 French was performed. Proceeding with the scope after dilatation, the prostatic urethra had an obstructive impression. The bladder wall showed trabeculae and pseudodiverticula - typical signs of chronic bladder outlet obstruction [11].

Two weeks after the intervention, voiding was described as improved but not satisfactory. Another urethrocystoscopy was not performed due to the patient's inability to tolerate local anesthesia. It was assumed that a recurrence of the urethral stricture two weeks after the dilatation was highly improbable. Considering the clinical context of the case, prostatic obstruction was identified as the underlying pathology for the residual bladder outlet obstruction.

Furthermore, significant RV in the urinary bladder remained after spontaneous voiding (spontaneous voiding portions: 20-160 ml, RV through suprapubic catheter: 30-150 ml). Maximal bladder capacity, which was calculated from the voided and residual volume, was 220 ml. IPSS was S=24 and L=6 [7]. Ultrasound showed a prostate volume of 60 cc. The patient requested further treatment to optimize voiding and to get rid of the suprapubic catheter. Pharmacological treatment with silodosin (Alpha-1-adrenoceptor antagonist) showed no improvement. In this case, we discussed different surgical techniques, which are recommended for moderate to severe LUTS due to prostatic enlargement.

TUR-P is a known and well-established procedure. Our research on PubMed^®^ showed publications from about five decades ago that described TUR-P through temporary perineal access to the urethra to prevent strictures of the anterior urethra [12,13]. However, access through a permanent perineostomy with modern instruments has not been described in the contemporary literature so far and neither performed at our clinic.

The preoperative assessment showed a prostate-specific antigen (PSA) of 2.76 µg/l. The digital rectal exam was unremarkable. Prostate Cancer (PCa) risk was assessed to be low, no further workup was performed. Preoperative urine culture showed growth of *Klebsiella pneumoniae.*

After explaining this premiere situation and the general risks of the intervention, the patient was willing to undergo surgery.

**Figure 2:**
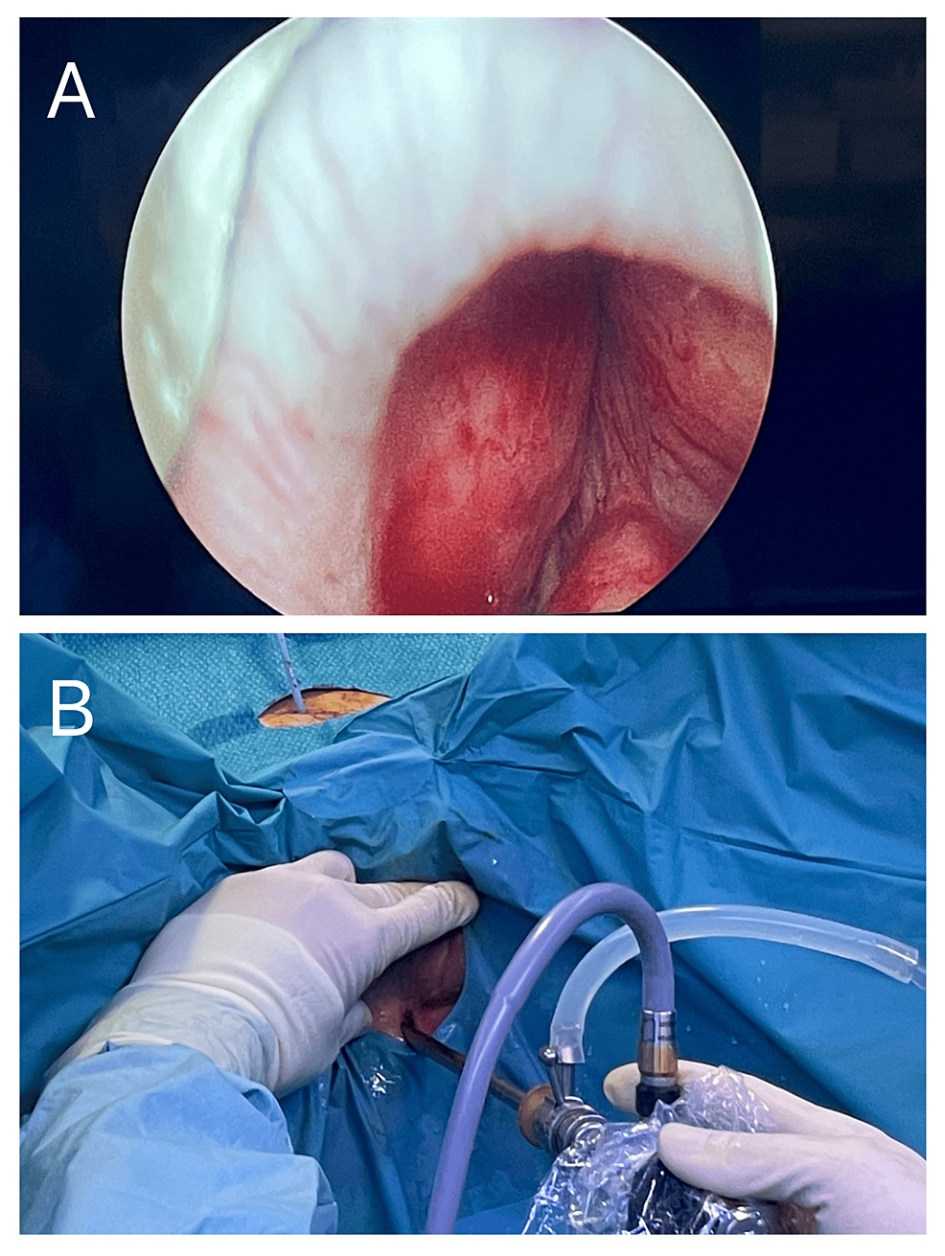
A) View of the prostatic urethra when entering with the resectoscope. Behind the external urethral sphincter (bright arc), the left and right lobes, and the seminal colliculus are shown. B) Setting of the access through the perineostomy with the resectoscope.

The surgery was performed in a lithotomy position under general anesthesia (Figure 2). Antibiotic treatment was started two days preoperatively. According to the European Association of Urologists guidelines, additional intravenous antibiotic treatment was given immediately before surgery [14]. Gyrus™ PlasmaKinetic System resectoscope was used (Olympus). Before inserting the resectoscope, the perineostomy was dilated up to 24 French, then, a resection plane between five and seven o'clock lithotomy position was created, followed by the resection of the middle lobe. Next, the forming of a groove at 5 and 7 o'clock with resection down to the prostatic capsule from the bladder neck to the colliculus was performed. Followed by ascending resection of the right and left side lobes and subsequently of the apical portion, according to the Mauermayer method [15,16]. The total of resected tissue was 18 grams, the estimated blood loss was 50 ml, and the resection time was 70 minutes. The suprapubic catheter was changed after the resection and an irrigation catheter was inserted through the perineostomy at the end of the procedure. Postoperative bleeding was minor, bladder irrigation was stopped on the first postoperative day. The irrigation catheter was removed on the second postoperative day and the patient was discharged with the remaining suprapubic catheter on the third postoperative day.

The first follow-up two weeks after surgery showed improved voiding with 70-80% through the perineostomy and 20-30% through the suprapubic catheter. In a follow-up visit six weeks later, the patient reported a satisfactory micturition situation with an IPSS of S=13 and L=2, which is a significant improvement of around 50% for the symptoms and >60% for satisfaction. No incontinence occurred. Uroflowmetry was not conclusive due to a voided volume of only 50 ml [17]. The bladder diary showed average voiding portions of 120 ml (n=12, range 100-150 ml) with an average RV of 15 ml (range 0-30ml). The patient felt confident with his bladder function. Therefore, the suprapubic catheter was removed. Seven weeks later, the patient reported bladder outlet obstruction symptoms again. Ultrasound showed 145 ml RV. The inspection of the perineostomy showed a stricture at the skin level. Another transperineal indwelling Foley catheter was guidewire-controlled inserted. Two months later, the stricture of the perineostomy was dilated with Optilume^TM^, a drug-coated (Paclitaxel) balloon. Intraoperative urethroscopy showed a widely open prostatic urethra. After that, RV was again significantly reduced to 10 ml. The follow-up until now (six months) was uneventful.

The histology report of the resected tissue showed PCa Gleason-Score 3+3=6, Grade Group 1. The case was presented at the interdisciplinary uro-oncological tumor board and active surveillance was recommended.

## Discussion

To our knowledge, after extensive literature research, there is no contemporary study available with a description of this procedure. Being aware that decades ago, perineal access to the urethra was formed to prevent iatrogenic urethral strictures, this temporary access lost attention in modern transurethral endoscopic surgery. Therefore, we consider this as the first reported case of a bipolar TPR-P through a permanent perineostomy. The procedure showed satisfactory outcomes with an absence of surgery-related adverse events.

At this point, we highlight the indication of the surgery. As described, at the time of presentation at the emergency room and the following visit, the main treatment focus was on the bulbar urethral stricture. After dilating this urethral obstruction, voiding symptoms and RV persisted. Due to the prostate enlargement, which was diagnosed while dilating the stricture, the indication for the surgery was given by the suspected prostatic bladder outlet obstruction. There was no uroflowmetry recorded nor urodynamic testing performed before the surgery.

Compared to the conventional TUR-P performed through a normal lengthy urethra, the presented approach demonstrated a notable increase in the range of motion of the resectoscope due to direct access to the membranous urethra. However, the shortened access pathway may diminish tactile feedback, typically essential for navigating the prostatic urethra and aligning the resectoscope with the prostate anatomy. On the other side, it could be assumed that this might be beneficial, especially for enucleation techniques such as endoscopic enucleation of the prostate. Additionally, for enhanced instrument control and protection of the external sphincter, we recommend using the non-dominant hand as a spacer between the instrument and the skin.

The minimal access perineal urethrostomy, described by Eylert and Bates [18], shows an example of using extra nephrolithotomy-like access through the perineum to gain extra length and maneuverability at the bladder neck while performing a holmium laser enucleation of the prostate. So this is an example of similar access but differs in some aspects. In our case, the perineostomy is a permanent solution of urinary diversion due to multiple penile debridements. Eylert and Bates formed the access at the same part of the urethra, though a minimally invasive and temporary version.

The resection time of 70 minutes was extended, compared to a standard TUR-P while the resected volume was low. In our case, this was due to several reasons. First, in addition to the prostate enlargement, the patient had a stricture, which needed dilatation prior to resection. Furthermore, the small bladder capacity resulted in frequent emptying of the bladder to prevent high pressure caused by the irrigation fluid. In addition, as a consequence of the diminished tactile feedback, the speed of the resection had to be adapted to not risk any injuries to the external sphincter.

The case demonstrates the treatment of a complex combination of several urologic problems such as strictured perineostomy, prostate enlargement, and small bladder capacity. Urethral strictures after perineostomy occur in 18% [19]. The patient had several risk factors for developing the mentioned stricture after the TPR-P such as UTI, catheterization, and instrumentation. However, the patient reported satisfaction after resolving the stricture of the perineostomy.

Post-voiding RV was reduced significantly. Due to the pre-existing small bladder capacity, the post-resection voiding volume did not exceed 150 ml. Due to limited uroflowmetry measurements, this report has limited objective data to comprehensively evaluate the outcome. Treatment success was therefore evaluated by voiding volumes, RV, and patient-related outcome measures such as IPSS.

Regarding preoperative PSA level and risk stratification through calculated PSA density and SWOP risk calculator [20], the probability of significant prostate cancer prediction was accurate. Incidental finding of ISUP grade 1 prostate cancer was managed with active surveillance.

## Conclusions

We report a case of transperineostomal resection of the prostate for prostatic bladder outlet obstruction.

Due to the missing full length of the urethra, the augmented range of motion seemed almost too loose for classic resection techniques. Therefore, we believe that in such cases, it might be an advantage to use enucleation techniques. However, in our case, TPR-P was feasible and safe with a good functional outcome.
